# It’s high-time to re-evaluate the value of induced-chemotherapy for reinforcing immunotherapy in colorectal cancer

**DOI:** 10.3389/fimmu.2023.1241208

**Published:** 2023-10-18

**Authors:** Shiya Yao, Yuejun Han, Mengxiang Yang, Ketao Jin, Huanrong Lan

**Affiliations:** ^1^ Department of Colorectal Surgery, Affiliated Jinhua Hospital, Zhejiang University School of Medicine, Jinhua, Zhejiang, China; ^2^ Department of Surgical Oncology, Hangzhou Cancer Hospital, Hangzhou, Zhejiang, China

**Keywords:** colorectal cancer, immunotherapy, inducing chemotherapy, immunogenic cell death, combination therapy, clinical trial, tumor microenvironment

## Abstract

Immunotherapy has made significant advances in the treatment of colorectal cancer (CRC), revolutionizing the therapeutic landscape and highlighting the indispensable role of the tumor immune microenvironment. However, some CRCs have shown poor response to immunotherapy, prompting investigation into the underlying reasons. It has been discovered that certain chemotherapeutic agents possess immune-stimulatory properties, including the induction of immunogenic cell death (ICD), the generation and processing of non-mutated neoantigens (NM-neoAgs), and the B cell follicle-driven T cell response. Based on these findings, the concept of inducing chemotherapy has been introduced, and the combination of inducing chemotherapy and immunotherapy has become a standard treatment option for certain cancers. Clinical trials have confirmed the feasibility and safety of this approach in CRC, offering a promising method for improving the efficacy of immunotherapy. Nevertheless, there are still many challenges and difficulties ahead, and further research is required to optimize its use.

## 1 Introduction

Colorectal cancer (CRC) is the third most common malignancy globally, after lung cancer, and is the second leading cause of cancer-related deaths in both men and women (1, 2). Despite improved screening for early detection, the global burden of disease and mortality has not significantly decreased (1). Approximately 20% of patients present with metastatic disease at diagnosis, and an additional 25% of patients who present with localized disease will subsequently develop metastases (3). Most patients with metastatic CRC (mCRC) cannot be cured and are managed with palliative systemic therapy, resulting in poor prognosis with a median overall survival (mOS) of approximately 30 months (3). In the past five years, immunotherapy has made a significant impact on the treatment of CRC, with immune checkpoint inhibitors (ICIs) being particularly prominent. The tumor immune microenvironment (TIME) is intimately linked with tumor immunotherapy and represents a key obstacle to successful antitumor immune therapy, potentially limiting its clinical benefit. Numerous studies have demonstrated the efficacy of ICIs in the treatment of microsatellite instability (MSI-H)/mismatch repair-deficient (dMMR) mCRC. However, 95% of mCRC patients are microsatellite stable (MSS)/proficient mismatch repair (pMMR) subtype and are insensitive to immune therapy (4, 5). We will discuss the reasons for poor response to ICIs in this patient population, including defects in antigen presentation and peptide transport, immune evasion, abnormalities in the TIME, low tumor mutation burden, and targeting of apoptotic pathways, among others. Efforts are currently underway to overcome these barriers and improve the sensitivity of immune therapy in this patient population.

It was previously believed that chemotherapy was solely an immunosuppressive agent. However, recent data indicate that chemotherapy drugs can promote immune activation through various pathways, notably via the induction of immunogenic cell death (ICD) mechanisms (6–9). Certain chemotherapy drugs, such as doxorubicin (DOX), paclitaxel (PTX), and oxaliplatin (OXA), can kill tumor cells via ICD, thereby activating innate and adaptive antitumor immune responses. ICD is characterized by the release of danger-associated molecular patterns (DAMPs) and the generation and processing of non-mutated neoantigens (NM-neoAgs) tumor-associated antigens to enhance antigen presentation by promoting dendritic cell (DC) maturation and cytotoxic T lymphocyte (CTL) infiltration (10). This process can reverse the tumor immune suppressive microenvironment and increase the sensitivity of immunotherapy. A series of clinical studies supporting the combination of chemotherapy and ICIs in mCRC is currently ongoing. Based on this, the concept of inducing chemotherapy has been introduced, which involves administering chemotherapy prior to immunotherapy to convert the tumor microenvironment (TME) from “cold” to “hot,” thereby enhancing the response to immunotherapy. Inducing chemotherapy combined with immunotherapy has become part of the standard treatment for certain cancers, with clinical trials confirming its feasibility in CRC. However, many challenges remain in the treatment of mCRC. The fusion of immunotherapy and Single-cell RNA sequencing (scRNA-seq) holds promise for providing truly personalized treatment for an increasing number of mCRC patients, enabling us to achieve personalized treatment strategies (11). Additionally, significant research is needed to optimize this combined treatment approach, including how to optimize dosage regimens, such as dose, timing, and sequence, and biomarker prediction studies. We look forward to further breakthroughs in the future to provide more effective and safer treatment options for cancer patients.

## 2 Exploring the progress and challenges of immunotherapy in CRC

Immune checkpoint inhibitors (ICIs) have brought new opportunities in cancer treatment (12–15). The programmed cell death protein 1/programmed death-ligand 1 (PD-1/PD-L1) signaling pathway in tumors is a crucial mechanism for evading immune surveillance. The FDA first approved the use of immunotherapy drugs for treating mCRC in 2017 (16–19). Pembrolizumab (an anti-PD-1 monoclonal antibody) has been established as the new standard for first-line treatment in MSI-H/dMMR mCRC (20).

**Figure 1:**
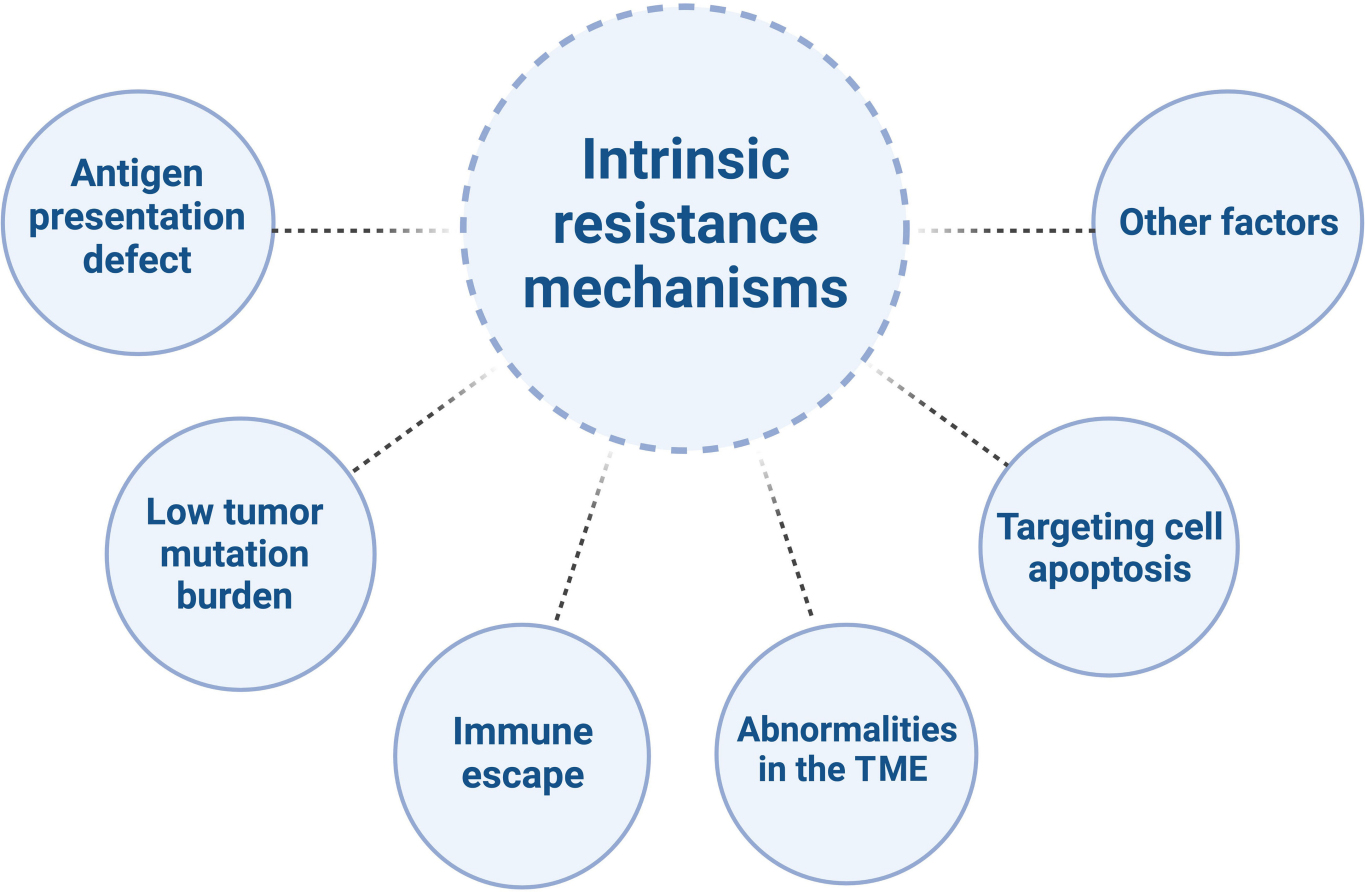
The intrinsic resistance mechanisms of MSS/pMMR mCRC to ICIs.

Despite the tremendous potential of immunotherapy in CRC treatment, there are also challenges and limitations. CRC is a common malignancy, with the majority being MSS/pMMR type. Compared to MSI-H/dMMR tumors, MSS/pMMR tumors have poorer response to immunotherapy, primarily due to immune suppression or immune desertification (21–23), characterized by low levels or defects in T-cell infiltration and reduced checkpoint protein expression (5). They generally do not benefit from immune therapies such as PD-1/PD-L1 inhibitors (24, 25), indicating obstacles to the effectiveness of immunotherapy (26). The mechanisms of resistance to immunotherapy in MSS/pMMR CRC are highly complex (**Figure 1**) (27).

Mutations in antigen presentation-related genes in tumor cells lead to loss of antigens within tumor cells, which makes it difficult for T cells to recognize and attack tumor cells, thereby reducing the sensitivity of tumor cells to immunotherapy. For example, the BRAF ^V600E^ mutation has been shown to reduce T cells infiltration into the TME and eliminate neoantigen presentation on cancer cells (28, 29). Inhibiting BRAF signaling has been demonstrated to reduce myeloid-derived suppressor cells, increase the recruitment of tumor-infiltrating lymphocytes, enhance neoantigen presentation on antigen-presenting cells, and collectively enhance anti-tumor immune responses (28, 30–32).

MSS/pMMR CRC tumor cells have a lower tumor mutation burden, which means that the effectiveness of immunotherapy is relatively poor. These types of tumors exhibit a relative deficiency in CD8+ T cell infiltration (33, 34), as well as lower tumor mutation burdens (35–38) and multiple immune antigen defects, leading to tumor immune evasion (39, 40). Immune escape refers to the ability of tumor cells to evade attacks from the immune system through various mechanisms (41). Immune escape mechanisms in MSS/pMMR CRC include the lack of immune stimulatory molecules, overexpression of immune inhibitory molecules, deficiency in major histocompatibility complex class I (MHC I) molecules, and lack of T cell infiltration (42–44). The activating mutations of KRAS, as an upstream regulator of BRAF and a potent activator of MAPK, may play a role in immune escape by impairing interferon-mediated antigen presentation and recruitment of effector T cells to the TME (45, 46). Another preclinical study showed that RAS oncogenes induce immune escape by stabilizing PD-1 RNA and leading to sustained expression of PD-1 (47). Increasing evidence suggests that the MAPK pathway may also be involved in immune exclusion, serving as another biological barrier to the success of immune therapy.

MSS/pMMR CRC is typically characterized by a “cold” or “excluded” TME, meaning that immune cells are unable to infiltrate the tumor or, even if they do, they are unable to exert their cytotoxic effects. Studies have shown that there is a reduced infiltration and activation of T cells in MSS/pMMR CRC, while the levels of immune suppressive cells (such as Tregs and myeloid-derived suppressor cells (MDSCs)) and immune inhibitory molecules (such as IDO1 and transforming growth factor-beta (TGF-β)) are elevated (48–50). This may weaken the anti-tumor response of T cells. For example, the increase in TGF-β is associated with an increase in Tregs, leading to downregulation of anti-tumor immunity. These data also support the role of the TGF-β pathway in downregulating NK cell activity, as NK cells play a role in innate immunity by recognizing cancer cells (51). It is worth noting that TGF-β activation has been observed in CRC liver metastasis, resulting in downregulation of CD4+ and CD8+ T cells (52). Activated B cells were found to be significantly depleted in liver metastases of CRC through scRNA-seq. The inhibitory effect on cancer cells was mediated by the suppression of the Wnt and TGF-β pathways through the SDF-1-CXCR4 axis, which promoted the migration of activated B cells (53).

In MSS/pMMR CRC, inhibition of the tumor cell apoptosis pathway renders the tumor cells insensitive to attacks from the immune system. Aberrant activation of the WNT/β-catenin signaling pathway is frequently observed in MSS CRC but is rare in MSI-H CRC (54, 55). Abnormal activation of the Wnt signaling pathway is associated with T cell exclusion and insufficient infiltration, which may lead to the inhibition of tumor cell apoptosis pathways and consequently result in treatment resistance (25, 54, 56–58). By inhibiting β-catenin pharmacologically, it is possible to increase the number of dendritic cells (DCs), upregulate CCL4, and promote the infiltration of CD8+ T cells in various tumor models, including CRC, thereby reactivating anti-tumor immune responses (59–61). Additionally, the overexpression of members of the Bcl-2 family and the presence of an immune suppressive microenvironment may also lead to the inhibition of tumor cell apoptosis pathways and consequently result in treatment resistance (62, 63). Other factors that may contribute to immunotherapy resistance in CRC include changes in the gut microbiome, which can impact the efficacy of immune therapy and lead to treatment resistance (64).

In summary, immunotherapy has had a profound impact on the traditional treatment of CRC, but it also poses challenges and limitations. Future research needs to further explore the combination of immunotherapy with conventional chemotherapy, overcome immune resistance mechanisms, and understand the influence of the TIME on the efficacy of immune therapy in order to enhance the effectiveness of immunotherapy and advance the treatment of CRC.

## 3 Mechanism of chemotherapy in promoting immune activation

Recent research in the field of immunotherapy has demonstrated that chemotherapy drugs not only enhance the immunogenicity of cancer cells but also induce immune stimulation by activating effector T cells and inhibiting immune suppressive cells. These exciting findings suggest that the combination of chemotherapy and ICIs may have synergistic anti-cancer effects, providing a promising treatment option for tumor patients who have a poor response to monotherapy with ICIs.

**Figure 2:**
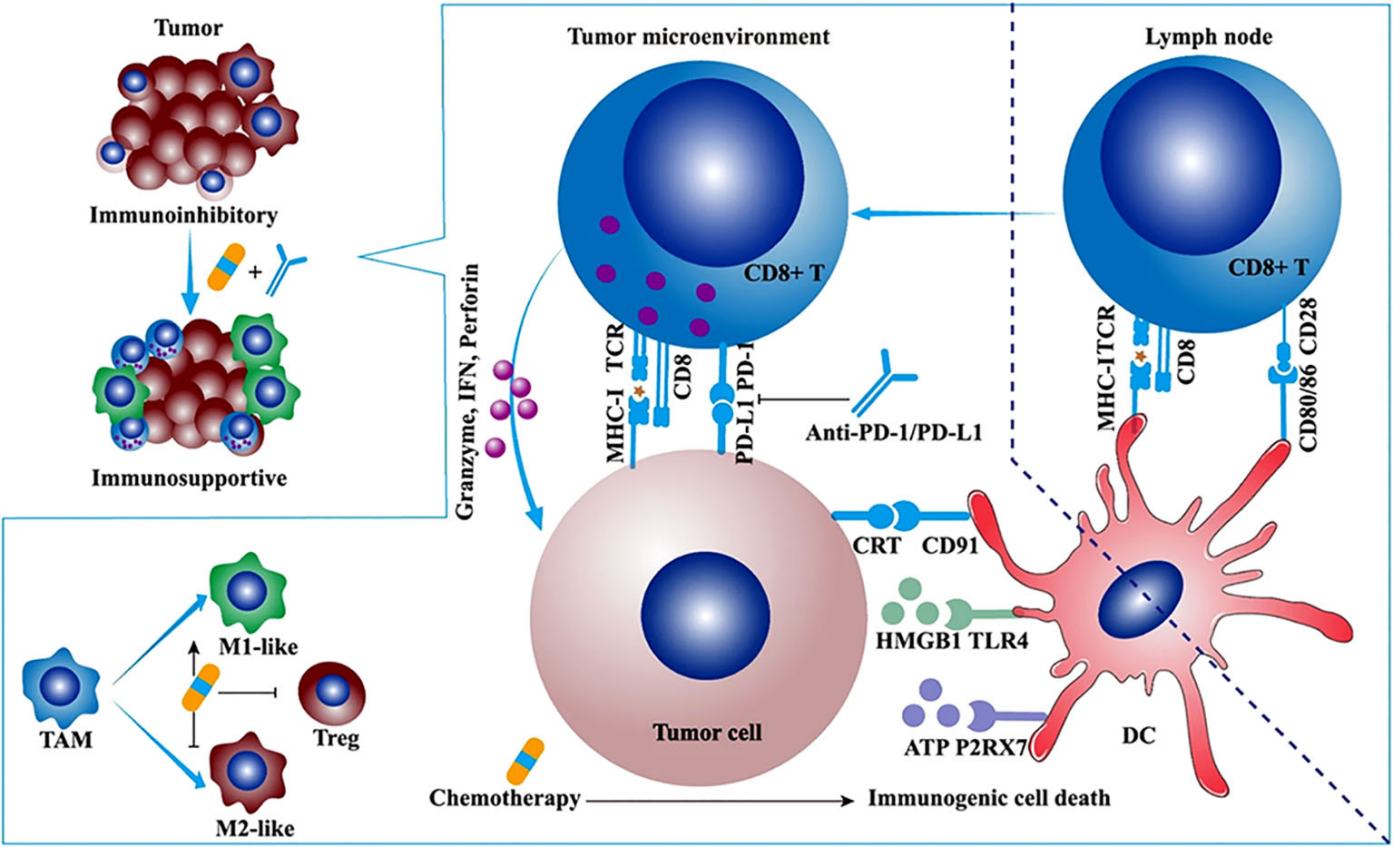
The synergistic antitumor efficacies and mechanisms of α-PD-1/PD-L1 in combination with chemotherapy, radiotherapy, or angiogenesis inhibitor. Chemotherapy synergizes with α-PD-1/PD-L1. Some cytotoxic chemotherapeutic drugs could induce immunogenic cell death and stimulate antitumor immune response. Immunogenic cell death (ICD) is featured with some upregulated damage-associated molecular patterns (DAMPs) such as calreticulin (CRT), ATP, and high-mobility group box 1 (HMGB1). The ATP-P2RX7, CRT-CD91, and HMGB1-TLR4 pathways facilitate the antigen capture and presentation of DC, ultimately motivating adaptive antitumor immune response. Apart from ICD, low-dose chemotherapy depletes regulatory T cells (Tregs) and promotes the repolarization of tumor-associated macrophage (TAM) from M2-like to M1-like phenotype (84).

Studies have shown that chemotherapy drugs may exert their effects through immune stimulation mechanisms (65). For example, anthracycline drugs can induce immunogenic cell death (ICD) and directly block immune inhibitory pathways in the TIME, leading to the release of neoantigens (NM-neoAgs) from cancer cells (6, 66–72). ICD is characterized by the presentation of dying cancer cells to antigen-presenting cells (APCs) in the form of danger-associated molecular patterns (DAMPs), which act as lymphoma adjuvant-like signals (73). Specifically, during the early stages of apoptosis, ICD induces the exposure of calreticulin (CRT) on the cell surface and releases extracellular ATP by upregulating autophagy during the detachment phase of apoptosis. It also promotes the release of high mobility group box 1 (HMGB1) during the secondary necrosis phase of cell death (71, 72, 74–82). CRT, ATP, and HMGB1 bind to their respective receptors (CD91 receptor, purinergic P2Y2 or P2X7 receptors, and TLR4) expressed on DCs, triggering their entry into the tumor tissue and upregulating antigen uptake processes. The resulting mature DCs (mDCs) present antigens and trigger a series of further immune responses. New antigens and danger signal molecules are released, forming a “cancer immune cycle” (83) (**Figure 2**). Overall, the ability of DCs to capture and present antigens is enhanced, and their ability to process NM-neoAgs and recruit CD4+ T cells/CD8+ T cells for an enhanced adaptive immune response in the tumor is more effective, thereby enhancing the immune system’s ability to clear cancer cells (66). Additionally, pro-inflammatory cytokines such as TNF, IL-6, and IL-1β, which are detected during and after ICD induction, can increase MHC I expression on APCs, promote T cell differentiation, and activate NK cells (85). Activated DCs (such as IL-12) and other innate immune cells (such as cytokines produced by IFN-α/β) enhance NK cell function, leading to the secretion of IFN-γ and TNF (86).

The TIME is a key barrier to anti-tumor immunity and may limit the clinical efficacy of immunotherapy (87, 88). Immune cells not only act as “gatekeepers” of the tumor but can also have positive or negative effects on tumor growth and metastasis (89, 90). For example, Tregs and MDSCs can suppress the immune response of T cells and natural killer cells (NK cells), providing favorable conditions for cancer invasion, inhibiting anti-tumor immune responses, and promoting metastasis (91, 92). TAMs can exhibit anti-tumor properties (93). When activated, TAMs can promote the proliferation and activation of anti-tumor T cells, thereby inhibiting tumor growth and metastasis (93).Although the number and function of NK cells may be suppressed, recent research has shown that certain drugs can enhance the function of NK cells and play a significant role in tumor treatment (94). Studies have shown that chemotherapy can eliminate specific cells, such as Tregs and MDSCs (95, 96), which have immunosuppressive characteristics. This can transform non-inflammatory tumors (referred to as “cold tumors”) into tumors rich in cytotoxic cells (referred to as “hot tumors”) (97), especially when used at doses below the maximum tolerated dose. Recent research indicates that anti-PD-1 therapy can reshape the tumor immune microenvironment based on chemotherapy-induced changes, providing new insights for improving the effectiveness of combination immunotherapy and chemotherapy (98).

**Figure 3:**
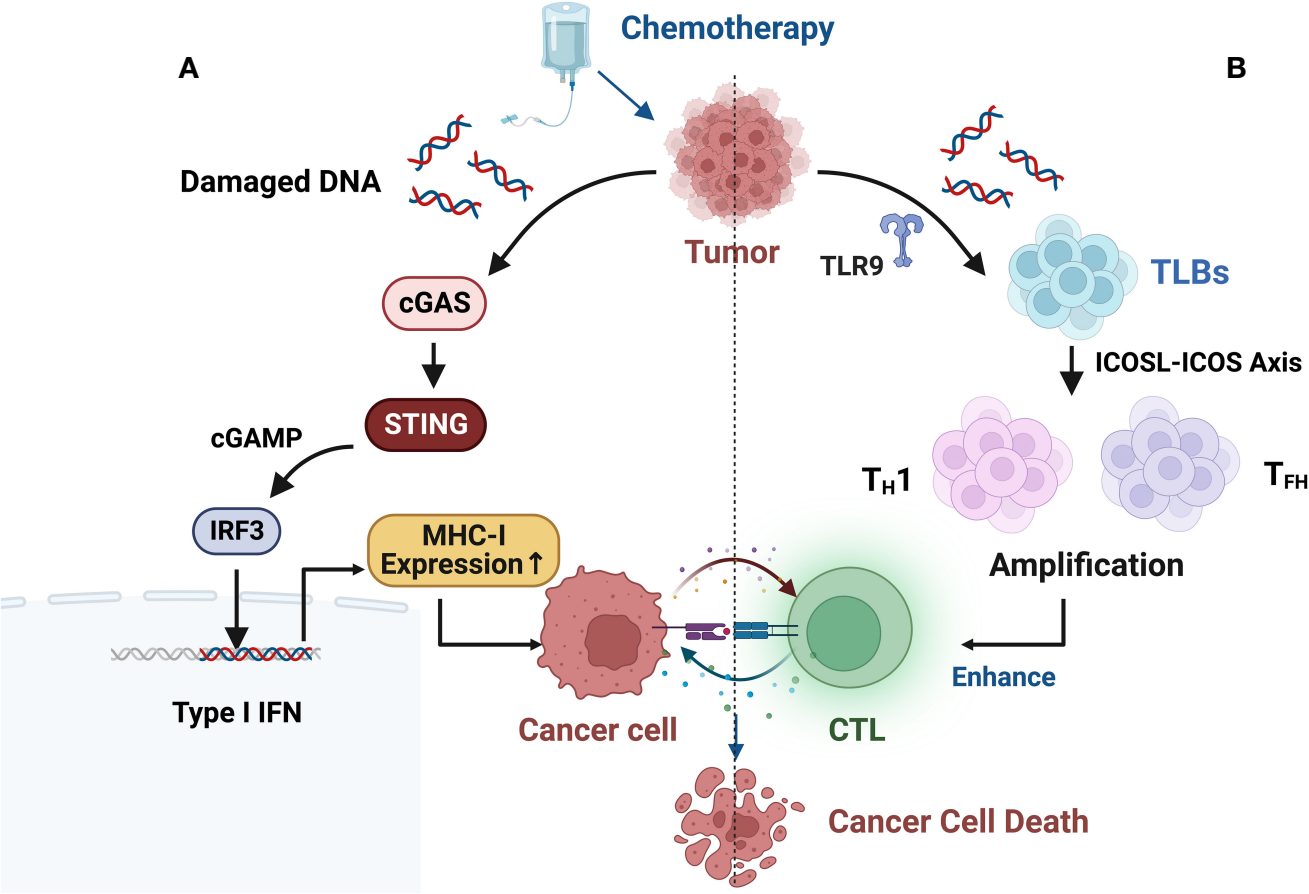
**(A)** Chemotherapy-induced DNA damage fragments in the nucleus may be actively exported to the cytoplasm to prevent their erroneous insertion into genomic DNA, thereby activating the innate immune cGAS-STING (cyclic GMP-AMP synthase/stimulator of interferon genes) pathway, resulting in an immune-rich microenvironment and triggering innate immune responses. **(B)** B cell-centered anti-tumor immune network. B-cell-centered anti-tumor immune network. Gemcitabine plus cisplatin (GP) chemotherapy activates an anti-tumor immune response dominated by a type of innate immune B cell (ILB). GP-mediated release of tumor cell DNA fragments can induce an ILB subset located in the third lymphoid structure induced by chemotherapy via Toll-like receptor 9 (TLR9) signaling. Subsequently, ILB promotes the expansion of type 1 helper T cells (T_H_1) and follicular helper T cells (T_F_H) via the ICOSL-ICOS signaling axis, thereby facilitating the cytotoxicity of CD8+ T lymphocytes (CTLs). Meanwhile, ILB can also activate the STING-IFN-I pathway of tumor cells, upregulating the expression of MHC I on tumor cells, forming a positive feedback loop.

Furthermore, some studies suggest that chemotherapy-induced DNA damage fragments in the cell nucleus may actively translocate to the cytoplasm to prevent their erroneous insertion into the genomic DNA. This process activates the innate immune cGAS-STING (cyclic GMP-AMP synthase/stimulator of interferon genes) pathway, leading to an immune-rich microenvironment and triggering innate immune responses (**Figure 3A**) (99–102).

Recent research has revealed new mechanisms of chemotherapy-induced immune modulation. Effective chemotherapy can induce B-cell-centered effector T-cell responses, suggesting that chemotherapy holds promising potential as a combination therapy with ICIs. This can be achieved by upregulating MHC I expression to directly modulate tumor immunogenicity and by enhancing the efficacy and quantity of CD8+ T cells through the enhanced interaction between endogenous-like B cells (ILBs) and effector helper T cells (T_H_ cells) (**Figure 3B**). ILBs enhance T_FH_ and T_H_1 cells via the ICOSL-ICOS axis. The inducible T-cell co-stimulatory (ICOS) pathway is another important pathway in tumor immunotherapy. ICOS is a co-stimulatory receptor expressed on activated T cells and is crucial for their survival and function. The ICOS pathway plays a critical role in balancing effector T cells and Tregs, and its dysregulation has been associated with the development and progression of various types of cancer (103). Lu et al. (104) also found that chemotherapy induces complement signaling pathways, enhancing the anti-tumor properties of B cells. Overall, these data suggest that in addition to therapeutic interventions targeting the restoration of conventional DC function, chemotherapy interventions can reshape the plasticity of B cells and establish an anti-tumor environment. Similar findings and breakthroughs are hoped to be achieved in CRC.

In conclusion, the mechanisms of immune modulation activated by chemotherapy provide new insights for the clinical treatment of combination immunotherapy. Further research is needed to explore the combined strategies of chemotherapy and immunotherapy in order to achieve better treatment outcomes and wider clinical applications.

## 4 Cytotoxic chemotherapy drugs that promote anti-tumor immunity

**Figure 4:**
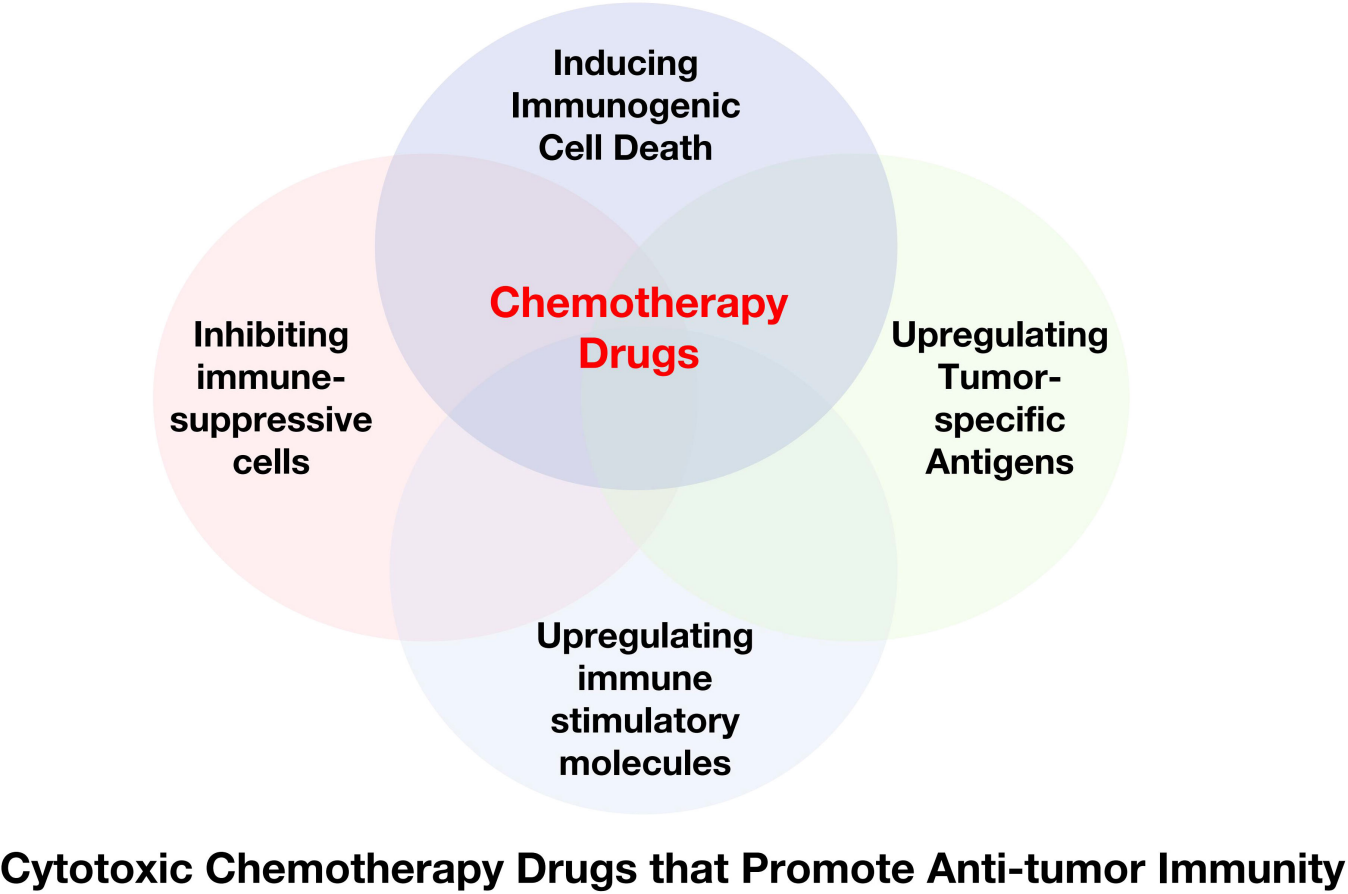
Cytotoxic Chemotherapy Drugs that Promote Anti-tumor Immunity. Some chemotherapy drugs have been shown to exert immune-stimulating effects by inducing immunogenic cell death, inhibiting immune suppressor cells, and/or activating effector cells, or upregulating tumor-specific antigens.

Chemotherapy drugs were initially designed to directly inhibit or kill malignant cells to achieve their therapeutic effect. Recently, some frontline drugs have been found to further promote anti-tumor immunity by increasing tumor immunogenicity, improving T cells infiltration, or depleting immune-suppressive populations (**Figure 4**). There is increasing evidence that chemotherapy triggers complex immune events (105–109), which is due to the ability of drugs to induce ICD in tumor cells and to directly modulate immune cells. Some chemotherapy drugs have been shown to exert immune-stimulatory effects by inhibiting immune-suppressive cells and/or activating effector cells, or by increasing immunogenicity and T cells infiltration (110–112). Chemotherapy drugs that promote anti-tumor immunity can be classified into several categories based on their mechanisms (**Table 1**).

**Table 1:** Immunological effects of conventional antitumor agents.

Agent	Effect	Notes	Reference
**Anthracyclines**	Inducing ICD	Activation of DCs and subsequent specific T cell responses	(113)
	Facilitating antigen presentation by DCs	Promoting the proliferation of CD8 +T cells of specific antigens in TDLNs and the infiltration of tumors by CD8+ T cells produced by IFN-γ	(114, 115)
**Cisplatin**	Inducing ICD	Increasing the infiltration of NK cells and local T cells oligoclonal dilation	(116)
	Inhibiting immune-suppressive cells	Consuming MDSCs and Tregs	(117)
	Upregulating the expression of immune stimulatory molecules	Recruitment of effector cells by upregulating MHC I expression of antigen-presenting cells	(118–120)
	Upregulating tumor-specific antigens	Immunosuppressive effect by upregulation of PD-L1	(121)
		Reducing IFN-γ production in T cells	(122)
**PTX**	Inducing ICD	Increasing the infiltration of NK cells and local T cells oligoclonal dilation	(116)
		B cells regulate antitumor CTL immunity	(104)
	Upregulating tumor-specific antigens	Promoting the proliferation of CD8+ T cells and T+H1 cells	(123–126)
	Inhibiting immune-suppressive cells	Consuming MDSCs and Tregs	(127, 128)
	Upregulating the expression of immune stimulatory molecules	Upregulating the expression of TAA and MHC I in tumor cells	(129)
**Etoposide**	Inducing ICD	Activating the IFN response of tumor cells	(130)
**Irinotecan**	Upregulating tumor-specific antigens	Upregulation of DAMPs and HMGB1 and HSP70	(131)
	Inhibiting immune-suppressive cells	Inhibiting Tregs proliferation and function	(132)
**Topotecan**	Upregulating tumor-specific antigens	Upregulation of MHC I and Fas expression	(85, 133)
**OXA**	Inducing ICD	Upregulation of DAMPs and HMGB1 and ATP	(76)
	Upregulating tumor-specific antigens	Inducing DCs to upregulate PD-L1 expression	(134)
	Upregulating the expression of immune stimulatory molecules	Increasing immune cells infiltration	(135)
**Gemcitabine**	Upregulating tumor-specific antigens	Inducing HLA1 expression	(136)
	Inhibiting immune-suppressive cells	Consuming MDSCs and Tregs	(137–139)
**5-FU**	Inducing ICD	Upregulation of DAMPs and HSP70 and ATP	(96, 140)
	Upregulating tumor-specific antigens	Promoting the maturation and functional enhancement of DCs	(141)
	Inhibiting immune-suppressive cells	Consuming MDSCs and Tregs	(96)
**Teniposide**	Upregulating tumor-specific antigens	Enhancing T cells recognition	(101)
**Dacarbazine**	Upregulating tumor-specific antigens	Leading to NK cells activation and release of IFN-γ	(142)
**CTX**	Inhibiting immune-suppressive cells	Inhibiting Tregs proliferation and function	(143, 144)
	Upregulating tumor-specific antigens	MHC I expression	(136)
**MTX**	Upregulating the expression of immune stimulatory molecules	Upregulating CD40, CD80 and CD83 to promote the maturation of DCs	(145)
**Docetaxel**	Inhibiting immune-suppressive cells	Consuming MDSCs and Tregs.	(67)

ICD, immunogenic cell death; TDLNs, tumor draining lymph nodes; MHC I, major histocompatibility complex class I;CTL, cytotoxic T lymphocyte; TAA, tumor-associated antigen; DAMPs, damage-associated molecular patterns; HLA1, human leukocyte antigen 1; HSP70, heat shock protein 70; ATP, adenosine triphosphate; MTX, methotrexate; CTX, cyclophosphamide; 5-FU, 5-fluorouracil; OXA, oxaliplatin; PTX, paclitaxel.

### 4.1 Inducing ICD

ICD is a form of apoptosis that can induce an effective anti-tumor immune response. Drugs that induce ICD include anthracycline chemotherapy drugs, OXA, and PTX (146–149). As mentioned earlier, these drugs induce an anti-tumor immune response by activating DCs and subsequent specific T cells responses.

Specifically, anthracycline chemotherapy drugs can induce ICD, which is a form of apoptosis that induces an effective anti-tumor immune response by activating DCs and subsequent specific T cells responses (150). Recent studies have found that drugs that induce ICD can also regulate anti-tumor CTL immunity through tumor-infiltrating NK and B cells. In human ovarian cancer, platinum-based and taxane-based chemotherapy significantly increased NK cells infiltration and local T cells oligoclonal expansion (116). In human breast cancer, a neoadjuvant regimen of DOX, cyclophosphamide(CTX), and PTX converted infiltrating tumor B cells to a new ICOSL+ phenotype. These newly appearing B cells participated in the formation of TLS and significantly increased the number and cytotoxicity of tumor-specific CD8+ T cells (104).Another topoisomerase II inhibitor, teniposide, can induce ICD, but its mechanism of action is different from that of anthracyclines. Topoisomerase II inhibitors induce proliferation arrest or death of tumor cells by increasing DNA double-strand breaks (130). As mentioned earlier, teniposide activates the endogenous type I interferon (IFN) response in tumor cells and upregulates features of ICD (**Figure 3A**). In murine colon cancer, teniposide induced potent anti-tumor CD8+ T cells immunity and significant tumor suppression. Administration of teniposide reversed the resistance of KRAS-mutant CT26 colon cancer to PD-1 blockade (151). Although it has positive immunomodulatory effects in mouse tumors, it remains unclear whether teniposide acts as an ICD inducer in human cancers. Considering its ability to activate anti-tumor CTL responses, chemotherapy drugs that induce ICD are thought to be able to enhance the therapeutic effect of ICIs. The combination of DOX and PD-1 or PD-L1 antibodies shows significant anti-tumor effects in various mouse cancers, such as melanoma and breast cancer (152, 153). In human metastatic triple-negative breast cancer (TNBC), short-term treatment with DOX induces sensitivity to PD-1 blockade (154). Similarly, OXA has been shown to enhance the anti-tumor effect of anti-PD-L1 therapy in mouse lung cancer, melanoma, and CRC (155). The combination of PTX and ICIs produce superior tumor suppression in non-immunogenic squamous non-small cell lung cancer (NSCLC) (156).

### 4.2 Upregulating tumor-specific antigens

Certain chemotherapy drugs can induce tumor cells to express antigens, thereby enhancing T cells recognition and killing of tumor cells.

For example, Irinotecan and Topotecan are derivatives of camptothecin, which can enhance T cells recognition of tumor cells and upregulate tumor-specific antigens (157). An *in vitro* experiment revealed the upregulation of DAMPs, HMGB1, and heat shock protein 70 (HSP70) after treatment with Irinotecan (131). In melanoma, they can upregulate tumor-specific antigens. Surviving tumor cells upregulate MHC I and Fas expression after treatment with Topotecan (85, 133), making them more susceptible to immune cells killing. PTX can stimulate DC maturation and antigen presentation through various mechanisms, such as the NF-κB and MAPK signaling pathways, TLR4/MyD88 pathways, etc. (158). In addition, PTX can promote an immune response of tumor-specific T cells (123–126) and promote the proliferation of CD8+T cells and T+H1 cells, thereby playing a role in the treatment of tumors. Platinum-based drugs and gemcitabine increase antigen specificity by inducing HLA1 expression (136). OXA can induce upregulation of PD-L1 expression on DCs, while carboplatin upregulates PD-1 mRNA expression. Studies have shown that in patients with head and neck squamous cells carcinoma receiving standard cisplatin treatment, cisplatin can have an immune suppressive effect through upregulation of PD-L1 (121). The expression of PD-L1 may also impede the response of anti-cancer T cells. *In vitro*, high-dose cisplatin significantly reduced IFN-γ production by T cells (122) and decreased the cytotoxicity of NK cells in ovarian cancer patients (159). Standard doses of 5-FU may produce an immune-stimulatory effect, for example, by promoting antigen uptake by DCs (141). DOX promotes antigen presentation by DCs, promotes the proliferation of CD8+ T cells specific for certain antigens in tumor-draining lymph nodes, and increases IFN-γ production by CD8+ T cells infiltrating the tumor (114, 115). Temozolomide enhances the tumor cells antigen presentation mechanism and enhances T cells recognition (101). Dacarbazine is currently only used in melanoma patients who are not eligible for new therapies or have failed other treatments. Dacarbazine can upregulate NK cells activation and IFN-γ release. Increased levels of IFN-γ lead to upregulation of MHC I expression in tumor cells, which is necessary for T cells recognition (142).

### 4.3 Inhibiting immune-suppressive cells

Certain chemotherapy drugs can inhibit the activity of immune-suppressive cells, thereby enhancing anti-tumor immune response. For example, platinum-based drugs, CTX, 5-FU, docetaxel, and other chemotherapy drugs can reduce the inhibitory effect of Tregs on the anti-tumor immune response. These chemotherapy drugs can also promote the polarization of tumor-associated macrophages, thereby enhancing their anti-tumor activity. Moreover, the combination of certain ICIs and chemotherapy drugs can further enhance the anti-tumor immune response.

Specifically, Platinum-based drugs reduce the immune-suppressive microenvironment by depleting MDSCs and Tregs (117). PTX can selectively inhibit the number and function of Tregs (114, 123, 124, 160–166). One study found that patients with advanced disease had a significant decrease in the anti-inflammatory cytokine IL-10 levels after receiving PTX treatment (167). PTX can also repolarize TAM2. It has recently been identified as an agonist for TLR4 on TAMs and directly polarizes this anti-inflammatory population towards a pro-inflammatory phenotype (168, 169). Breast cancer patients treated with PTX exhibit peripheral pro-inflammatory features (170). After PTX treatment, ovarian cancer patients have gene enrichment associated with the inflammatory macrophage phenotype (168). Studies have shown that the combination of atezolizumab and nab-PTX prolongs progression-free survival (PFS) in patients with metastatic TNBC (171, 172). Low-dose CTX not only reduces the number of Tregs in tumors but also inhibits Tregs function (143). A recent study found that CTX preferentially targets CCR2+ Tregs in a highly active and proliferative state, namely effector Tregs (173). A clinical trial also showed that repeated low-dose CTX induction of Tregs depletion and enhanced anti-tumor immunity in patients with end-stage metastatic CRC ultimately contributes to prolonged progressive survival (174). CTX can also deplete tumor-infiltrating Tregs and improve the survival rate of mice with neuroblastoma when used in combination with anti-PD-1 therapy (144). Similar to CTX, topoisomerase I inhibitor camptothecin can also inhibit the production and function of Tregs. By removing the suppression of Tregs, irinotecan promotes the initiation and proliferation of CD8+ T cells in draining lymph nodes and inhibits the growth of lung cancer and CRC in a CD8+ T cell-dependent manner (175). Similarly, it has been reported that the FOLFIRI chemotherapy regimen containing irinotecan can reduce the inhibitory activity of peripheral Tregs in CRC patients (132). 5-FU selectively kills MDSCs *in vivo* while preserving other lymphocyte subtypes (96). Gemcitabine can deplete circulating or tumor-infiltrating MDSCs in various cancers, which benefits the restoration of CTL infiltration and cytotoxic activity (67). The use of standard doses can reduce the number of MDSCs while enhancing the cross-presentation of malignant antigens (136). In pancreatic cancer patients, standard-dose gemcitabine leads to the depletion of Tregs (139). Interestingly, there is no significant decrease in other lymphocyte subtypes after treatment.

### 4.4 Upregulating immune stimulatory molecules

Certain chemotherapy drugs can upregulate the expression of immune stimulatory molecules, thereby enhancing anti-tumor immune response. For example, PTX and its analogs can upregulate the expression of TAA and MHC I on tumor cells (129).

High-dose methotrexate can cause bone marrow suppression (176), but low-dose methotrexate exhibits immune stimulatory properties (145). In an *in vitro* experiment, non-cytotoxic low-dose methotrexate concentrations promoted DC maturation by upregulating CD40, CD80, and CD83 (145). In turn, DCs stimulated T cells proliferation, which may lead to a stronger anti-tumor response. This suggests that low-dose methotrexate can be used as an immune stimulant. CTX can induce MHC I expression (136) and deplete Tregs cells (114). Cisplatin also exhibits immune stimulatory properties by upregulating MHC I expression on antigen-presenting cells (118, 119), recruiting effector cells to the tumor site, and stimulating their proliferation (120). A single dose of OXA increased immune cell infiltration in a CRC mouse model (135). In ovarian cancer, a single dose of gemcitabine increased CD8+ T cell tumor infiltration and PD-L1 expression both *in vivo* and *in vitro* (139, 177).

## 5 Clinical application of the combination of chemotherapy and immunotherapy in CRC

As previously discussed, chemotherapy can activate immune regulation through various mechanisms, thereby enhancing patients’ response to immunotherapy. Combination immunotherapy has become an effective strategy for treating certain tumors. Therefore, combining chemotherapy and immunotherapy may be a new treatment strategy. In fact, some studies have already demonstrated the clinical efficacy of this combination strategy. For example, in first-line treatment for NSCLC, Pembrolizumab in combination with chemotherapy has been approved for first-line treatment of advanced non-squamous NSCLC regardless of PD-L1 levels (178). Other promising combinations include Atezolizumab, carboplatin, and etoposide for small cell lung cancer (SCLC), and nab-PTX or PTX in combination with Atezolizumab for advanced/metastatic breast cancer (179). Combination chemotherapy and immunotherapy have been shown to significantly improve patient survival. However, due to tumor heterogeneity and immune escape, a subset of patients with CRC lack response to immunotherapy. Currently, a series of clinical trials are being conducted for the combination of chemotherapy and immunotherapy in MSS/pMMR mCRC, with the aim of finding a breakthrough in treatment for these patients.

FOLFOX plus bevacizumab is the first-line standard of care (SOC) for MSS mCRC. The Checkmate 9X8 study (180) challenged first-line treatment of mCRC with the combination of nivolumab plus mFOLFOX6 and bevacizumab versus mFOLFOX6 and bevacizumab, with 95% of the patients being MSS/pMMR. The phase II results showed that, compared to the control group (current standard treatment regimen), the experimental group had a higher PFS rate starting at 12 months, with significantly improved 15-month PFS rate (45% *vs*. 21.5%) and 18-month PFS rate (28% *vs*. 9%), and ORR increased from 46% to 60%.

The BACCI phase II trial (181) (NCT0287319) evaluated the efficacy of adding Atezolizumab to Capecitabine and Bevacizumab in refractory mCRC. The addition of Atezolizumab to Capecitabine and Bevacizumab significantly extended progression-free survival (PFS), demonstrating a positive research advancement. This is the first positive study targeting the PD-1/PD-L1 pathway, chemotherapy, and the VEGF pathway, highlighting the need for further analysis and research.

The domestic BBCAPX study (182) is a study of the first-line treatment of MSS/RAS mutation mCRC with sintilimab + CapeOX + bevacizumab. The phase II single-arm trial results showed an ORR of up to 84%, a DCR of 100%, and unexpected conversion to R0 resection in 3 cases (12%). The study results demonstrated that the combination of sintilimab with CapeOX and bevacizumab for the treatment of RAS gene mutations and MSS-type mCRC showed good clinical benefits, with a high objective response rate and unexpected conversion rate, as well as low toxicity and tolerable safety. Based on the results of this phase II study, the ongoing BBCAPX phase III study holds great promise.

The objective of the single-arm phase II MEDITREME trial was to (183) evaluate the efficacy of the combination treatment with pembrolizumab, tremelimumab, and mFOLFOX6 in patients with MSS mCRC. The study results showed that the combination treatment resulted in a 3-month PFS rate of 90.7%, an overall response rate (ORR) of 64.5%, a median PFS (mPFS) of 8.2 months, and overall survival (OS) has not been reached yet.

NIVACOR (NCT04072198) (184) is a single-arm, open-label, multicenter phase II study with a safety assessment phase. Eligible patients with KRAS/BRAF-mutated metastatic CRC can participate and receive first-line treatment. Patients will receive FOLFOXIRI/Bevacizumab in combination with Nivolumab as induction therapy every two weeks, followed by maintenance therapy. Preliminary safety results indicate that this combination regimen is generally well-tolerated with acceptable toxicities. There is a high expectation for positive outcomes.

A study (185) evaluated the efficacy of PD-1/PD-L1 inhibitors in combination with the OXA-fluorouracil-leucovorin (mFOLFOX6) regimen in 30 patients with unresectable mCRC. The results showed a disease stabilization rate of 100% at 8 weeks and an overall response rate of 53% at 24 weeks. OXA and 5-FU led to increased ICD and antigen presentation. The study emphasized the potential benefits of combining chemotherapy with ICIs, as the combination of mFOLFOX6 and anti-PD-1 therapy was within an acceptable toxicity profile. The results showed that ICIs should be given concurrently or early after FOLFOX treatment and demonstrated clinical efficacy in pMMR CRC patients, showing promising results in patients with unresectable CRC.

Chemoradiotherapy also plays an important role in enhancing tumor response to immunotherapy. Current research indicates that radiation therapy can increase the expression of antigens on tumor cells, enhance tumor cell immunogenicity, and promote immune cell infiltration (186, 187). Therefore, combining immunotherapy with radiotherapy may lead to better therapeutic outcomes. For example, Lin et al. (188) used short-course therapy combining radiotherapy, sequential immunotherapy, and chemotherapy to treat CRC patients, which showed a pCR rate of up to 48%. In addition, the ongoing TORCH trial (189) is using toripalimab in combination with chemoradiotherapy or CapeOX in MSS CRC patients, with a proportion of 81.3% achieving cCR or pCR. These results suggest that combining immunotherapy with chemoradiotherapy may be an effective option for the treatment of CRC patients.

Most immunogenic chemotherapy agents have been shown to evoke immune stimulation not only by increasing the immunogenicity of cancer cells, but also by activating effector T cells and suppressing immune suppressor cells. These results suggest that the combination of chemotherapy and ICIs can have a synergistic anticancer effect and indicate that chemotherapy in combination with immunotherapy may be suitable for tumors that respond poorly to ICIs monotherapy. There are also ongoing prospective studies whose safety has been proven feasible, and the results of which are highly anticipated.

## 6 Preclinical and clinical studies of inducing chemotherapy combined with immunotherapy

Considering the immune-activating effects of chemotherapy drugs, the combination of chemotherapy and ICIs is an appropriate partner to achieve rapid and long-term cancer control. Based on these findings, we propose the concept of inducing chemotherapy, which involves using immunogenic chemotherapy drugs to change the timing before immunotherapy, converting “cold” tumors into “hot” metastases to initiate or restore anti-tumor immune responses, thereby enhancing the efficacy of ICIs (190). Some preclinical studies are being conducted in targeted preclinical models of CRC.

Song et al. (155) investigated the efficacy of OXA and anti-PD-L1 drugs in a microsyngeneic transplantation mouse model based on the CT26 cell line and found that the combination therapy of OXA and anti-PD-L1 drugs significantly slowed tumor growth compared to the use of OXA alone (191).

Dosset et al. conducted an interesting preclinical study using a microsyngeneic mouse model of two MSI-H CRCs (CT26 and MC38) and observed that adding adjuvant ICIs after FOLFOX could induce complete and durable tumor responses, whereas FOLFOX or ICIs alone were ineffective (192). Therefore, adding ICIs enables CD8+ T cells recruited by FOLFOX to induce effective anti-tumor immune responses (140). This is the first description of the PD-1/PD-L1 pathway as part of FOLFOX chemotherapy-induced adaptive immune resistance, indicating that chemotherapy can enhance the efficacy of ICIs (192). This correlation can attract a population of effective T cells which creating a favorable environment for immunotherapy to work. It has been shown to be associated with improved patient survival, especially after the emergence of immunotherapy (193).

The successful outcomes achieved in these preclinical trials provide strong evidence for the future implementation of clinical trials involving inducing chemotherapy (**Table 2**). Studies have shown that inducing chemotherapy has become one of the standard treatment options for certain tumors. For example, the combination of pembrolizumab with platinum and 5-FU was recently approved for metastatic and recurrent head and neck cancer based on its OS benefit (197).

**Table 2:** Summary of clinical trials and preclinical studies of chemotherapy combined with immunotherapy.

Summary of Clinical Trial Results of ICIs Combined with Chemotherapy in CRC											
Study	Patient characteristics	Treatment		Expected outcome						NCT Identifier	Reference
				median PFS (mo)		OS (12 mo)		ORR (%)	DCR (%)		
**BACCI**	pMMR/MSS	Cap, 850 or 1000 mg/m^2^ D1-14, Bev, 7.5 mg/kg D1, Atezo, 1200 mg D1 in 21 D cycles.		ArmA	4.4	43%		4.35	N/A	NCT02873195	(181)
	Arm A, 46 pts					52%		8.54			
	Arm B, 82 pts			ArmB	3.3	N/A		8.54			
**CheckMate 9X8**	180 untreated pts with mCRC	Nivo, 240 mg every 2w + mFOLFOX/Bev every 2w		1.9		N/A		60	91	NCT03414983	(180)
		mFOLFOX/Bev every 2w						46	84		
**BBCAPX**	25 unresectable, RAS-mutated, MSS mCRC	Sintilimab (200mg, D1) +Cap (1 g/m^2^, bid, D1-14) OXA (135 mg/m^2^, D1) + Bev (7.5 mg/kg, D1), in 21 D cycles.		N/A		N/A		84	100	NCT05171660	(182)
**MEDITREME**	57 cases of unresectable RAS mutant mCRC	Dur (750 mg, once every 2w) + Tre (75 mg, once every 4w) + mFOLFOX6. Pts with SD or PD: Dur (750mg, once every 2w) for maintenance		8.2		N/A		64.5	N/A	NCT03202758	(183)
**NIVACOR**	73 untreated pts with advanced RAS/BRAF-mutated mCRC	Nivo 240 mg, Bev 5 mg/kg + FOLFOXIRI administered every 2w for a total of 8 cycles.		10.1		N/A		76.7	N/A	NCT04072198	(184)
Summary of Clinical Trial Results of ICIs Combined with Radiotherapy in LARC Studies											
Study	Patient characteristics	Treatment		Expected outcome						NCT Identifier	Reference
**TORCH**	130 LARC	Arm A: SCRT (25 Gy/5Fx) + 6 cycles ToriCAPOX. Arm B: 2 cycles ToriCAPOX+ SCRT+ 4 cycles ToriCAPOX.		**MSS**						NCT04518280	(189)
				**pCR**			**cCR**				
				72.73%			81.25%				
Preclinical Studies of Inducing Chemotherapy Combined with Immunotherapy in CRC											
Experimental subjects		Experimental drugs		Outcome							Reference
CT26 micro-allotransplanted mice		OXA+ engineered PD-L1 trap		Combination therapy slowed tumor growth.							(155)
CT26 and MC38 mouse models		FOLFOX+ anti-PD1 blocking antibody		FOLFOX activated tumor-specific PD-1 CD8 + T cells in TME.							(192)
Clinical trials combining chemotherapy induction with immunotherapy in CRC											
Patient characteristics		Treatment	Outcome							NCT Number	Reference
Cohort A:37 mCRC		Cohort A: FTD/TPI 35 mg/m^2^ (bid, D1-5) + OXA 85 mg/m^2^ and Bev 5 mg/kg (D1). Cohort B: Nivo 3 mg/kg.	Increase of enzyme granules B, IFNγ and TNFα, upregulation of PD-L1 and PD-1 expression							NCT02848443	(194)
Cohort B: 17 MSS mCRC											
MSS mCRC with SD or PD on prior fluoropyrimidine-based therapy		Cap 1000 mg/m^2^ po bid D1-14 Q21 D (confirmed RP2D) + Pem 200 mg IV D1 Q21D + Bev 7.5 mg/kg IV D1 Q21 D	ORR: 5% mPFS: 4.3 m, mOS: 9.6 m							NCT03396926	(195)
MSS, MGMT silent unresectable mCRC		Phase I: Tem 150 mg/sqm po, D1-5, q4w for two cycles; Phase II: Tem 150 mg/sqm po, D1-5, q4w, + Nivo 480 mg q4w + ipi 1 mg/kg q8w.	mPFS: 7.0m, mOS: 18.4m, ORR:45%							NCT03832621	(196)

Atezo, atezolizumab; Bev, bevacizumab; Cap, capecitabine; Pem, pembrolizumab; Dur, Durvalumab; Tem, Temozolomide; Tre, Tremelimumab; Ipi, ipilimumab; CRC, colorectal cancer; DCR, disease control rate; MSI-H, microsatellite instability-high; pMMR, proficient DNA mismatch repair; MSS, microsatellite stable; Nivo, nivolumab; N/A, not available; NCT, National Clinical Trial; ORR, overall response rate; OS, overall survival; PFS, progression-free survival; 5-fluorouracil, 5-FU; OXA, oxaliplatin; mo, month; pts, patients; D, day; w, week; LARC, Locally advanced rectal cancer; pCR, pathological complete response; cCR clinical, complete response; ToriCAPOX, Toripalimab plus capecitabine and oxaliplatin; SCRT, short-course radiotherapy; SD, stable disease; PD, progressive disease; RP2D, recommended phase II dose; mDOR, median Duration of Response; TME, tumor microenvironment; FTD/TPI trifluridine/tipiracil; IV, intravenous; ivgtt, intravenously guttae; FOLFOX: OXA: 100 mg/m^2^ D1; Tetrahydrofolate: 200 mg/m^2^ ivgtt D1-5; 5-Fu: 500 mg/m^2^ ivgtt D1-5.

mFOLFOX6: Leucovorin, 400 mg/m^2^ D1; OXA, 85 mg/m^2^ D1, and 5-FU (400 mg/m^2^ bolus and then 2, 400 mg/m^2^ over 46 hours).

Ma et al. (198) presented the results of a phase III multicenter randomized controlled clinical trial on sequential treatment of locally advanced nasopharyngeal carcinoma with PD-1 inhibitor sintilimab and concurrent chemoradiotherapy after inducing chemotherapy at the 2023 American Society of Clinical Oncology (ASCO) Annual Meeting. Patients were randomly assigned to two groups, one receiving standard GP inducing chemotherapy and concurrent chemoradiotherapy, and the other adding sintilimab to the standard treatment. The primary endpoint was event-free survival (EFS). From December 2018 to March 2020, a total of 425 patients were recruited, and after a median follow-up of 42 months, sintilimab increased the 3-year EFS rate from 76% to 86%, a 10% improvement, and reduced the risk of relapse, metastasis, and death by 41%. The risks of local-regional recurrence and distant metastasis were reduced by 48% and 43%, respectively. This trial is the first to achieve a positive EFS result in all locally advanced head and neck cancers, demonstrating the feasibility of inducing chemotherapy as a promising strategy to optimize anti-tumor treatment. In addition, concurrent chemoradiotherapy after inducing chemotherapy is the standard treatment for locally advanced nasopharyngeal carcinoma. Zhang et al. (199) conducted a multicenter randomized trial, assigning patients to receive concurrent chemoradiotherapy or GP inducing chemotherapy followed by concurrent chemoradiotherapy. The median follow-up time was 69.8 months, and the 5-year OS rate in the inducing chemotherapy group was significantly higher than that in the concurrent chemoradiotherapy group (87.9% *vs*. 78.8%), with equivalent risks of late toxicities (≥grade 3) (11.3% *vs*. 11.4%). This study suggests that inducing chemotherapy before concurrent chemoradiotherapy can significantly improve the OS of patients with locally advanced nasopharyngeal carcinoma without increasing the risk of late toxicities.

Based on the effective results obtained from preclinical trials, a series of clinical trials combining chemotherapy induction with immunotherapy have also been conducted in CRC, and significant progress has been achieved.

**Figure 5:**
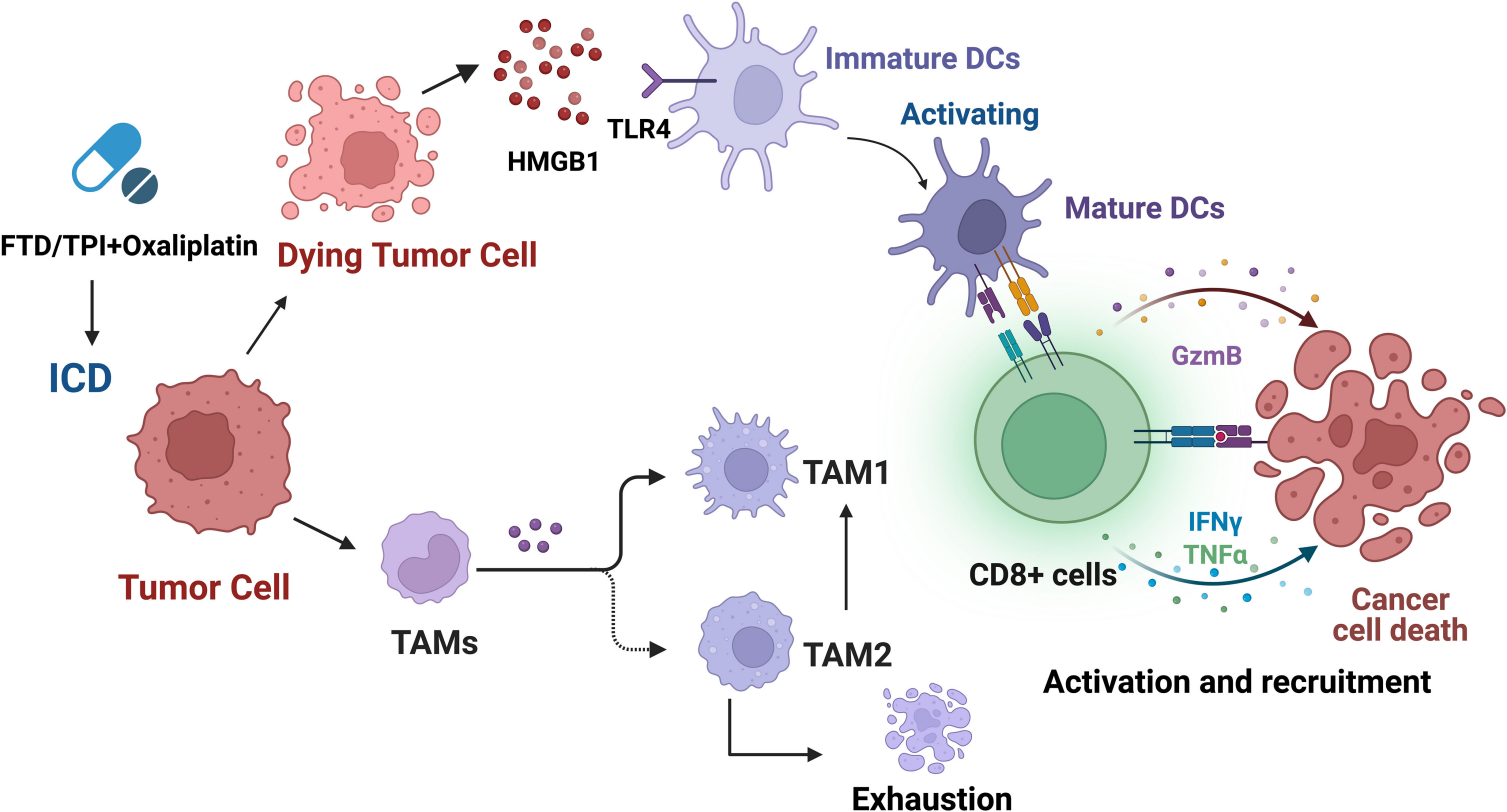
Trifluridine/tipiracil (FTD/TPI), oxaliplatin, or their combination not only induces ICD but also leads to enhanced infiltration and activation of cytotoxic CD8+ T cells, and increases the production of granzyme B, IFNγ, and TNFα in CD8+ T cells within the tumor. In addition, it leads to TAM depletion, especially TAM2, resulting in changes in the TAM1/TAM2 ratio, and upregulation of PD-L1 and PD-1 expression.

According to studies, TAM depletion induced by trifluridine/tipiracil (FTD/TPI), OXA, or combination therapy, especially TAM2, results in changes in the TAM1/TAM2 ratio, as well as enhanced infiltration and activation of cytotoxic CD8+ T cells, enhanced production of granzyme B, IFNγ, and TNFα in CD8+ T cells within the tumor, and upregulation of PD-L1 and PD-1 expression (**Figure 5**) (194). The combination use of FTD/TPI and OXA also induces ICD *in vivo*, providing a basis for using these drugs to eliminate immune-suppressive cells and improve checkpoint efficacy in patients with metastatic MSS CRC. The combination of FTP/TPI and OXA has been shown to be safe and effective in a phase I human clinical trial (200).

A phase II clinical trial (195) is currently ongoing to evaluate the safety, tolerability, and initial efficacy of pembrolizumab in combination with capecitabine and bevacizumab for the treatment of MSS mCRC patients. Bevacizumab, capecitabine, and pembrolizumab are used for treatment in the trial. The study results showed that the ORR among 40 evaluable patients was 5%, with a mPFS of 4.3 months and a mOS of 9.6 months. It is worth noting that MSS mCRC is rarely responsive to monotherapy with pembrolizumab, but capecitabine and bevacizumab may promote immune stimulation. These results suggest that the combination of pembrolizumab with capecitabine and bevacizumab may have some efficacy for MSS mCRC patients. However, it is important to note that the size of this trial is smaller, and further research and large-scale Phase III trials are needed to confirm the effectiveness and safety of this treatment regimen.

The MAYA II phase clinical trial (NCT03832621) (196) studied the combination of Nivolumab, Ipilimumab, and Temozolomide (TMZ) for the treatment of MSS, MGMT silenced unresectable mCRC patients who have not progressed, regardless of RAS mutational status. In the pre-selected 716 patients, 33 patients (24%) achieved disease control, which represents the final study population. The mPFS was 7.0 months, the mOS was 18.4 months, and the ORR was 45%. A series of temozolomide initiation followed by low-dose ipilimumab and nivolumab combination may induce durable clinical benefits in MSS and MGMT silenced mCRC. The initiation of treatment with tremelimumab provides the basic principle for immune sensitization induced by hypermutation in pMMR/MSS (MGMT-silenced) mCRC.

A randomized phase II trial (201) evaluated the safety of immunotherapy in combination with SOC in untreated MSS mCRC patients. Patients were randomized to receive SOC alone or SOC plus immunotherapy, which included mFOLFOX6 + Bevacizumab with or without AdCEA vaccine and Avelumab. In this small, randomized trial, the addition of immunotherapy did not significantly improve mPFS or overall response rate (ORR) compared to SOC alone. However, the SOC + immunotherapy regimen yielded biological activity in the form of substantial increases in multifunctional CD4+ and CD8+ T cells specific for the cascade antigens MUC1 and brachyury. Among them, the MUC1 and Brachyury pathways play important roles in cancer development and immune evasion and have become potential targets for tumor immunotherapy (202, 203).

A study (204) aimed to evaluate the safety, activity, and biomarker patterns of FOLFOX treatment with atezolizumab (anti-PD-L1) and bevacizumab (anti-VEGF-A) in patients with MSS mCRC. As of September 1, 2015, 52% of patients showed RECIST responses, with a mPFS time of 14.1 months and a median response duration of 11.4 months. No unexpected toxicities were observed. Wallin et al. found an increase in the expression of CD8+ T cells and PD-L1 in tumors after FOLFOX treatment alone and after combined FOLFOX, atezolizumab, and bevacizumab treatment. In some patients’ tumors, there was also an increase in cytotoxic T-cell markers (such as IFN-γ, GZMB, EOMES). Patients with increased tumor-infiltrating CD8+ T cells, which were consistent with increases in cytotoxic T-cell markers and PD-L1 expression, showed sustained responses or long-term disease control. These data further confirm that the combination of FOLFOX, atezolizumab, and bevacizumab may promote immune-related activities in CRC, thereby enhancing efficacy.

Although inducing chemotherapy combined with immunotherapy has achieved significant results, studies have shown that some chemotherapy drugs exhibit different immunogenic effects depending on their regimen, timing, dose, or administration sequence, even when used in combination with ICIs. One study investigated the impact of drug administration sequence and found that CTX given one day before anti-CTLA-4 therapy resulted in immune-mediated anti-tumor responses. However, when the sequence was reversed, CD8+ T cells underwent massive apoptosis, and the anti-tumor effect of anti-CTLA-4 was weakened (205). Another study tested three different regimens in NSCLC patients: a phase II study evaluating chemotherapy given before ipilimumab, a concurrent regimen, and a control group receiving placebo and chemotherapy (206). The primary endpoint of improved PFS was only achieved in the sequential regimen. A study investigated the effect of various types of chemotherapy on the treatment of metastatic triple-negative breast cancer (TNBC) and found that 2 weeks of low-dose chemotherapy during the induction period was more effective than nivolumab monotherapy (207). In addition, compared to the no-induction period, the number and clonality of T cells in the tumor were higher after chemotherapy-induced treatment (208).

Excitingly, inducing chemotherapy has become one of the standard treatment options for certain tumors, and a series of clinical trials on inducing chemotherapy have been conducted in CRC, achieving promising results. We propose the exploration of personalized treatment plans, gradually reducing the chemotherapy regimen and combining it with immunotherapy after inducing chemotherapy, until only oral chemotherapy is maintained, and eventually achieving maintenance therapy with a single immunotherapy. We look forward to further confirmation and application in the future.

## 7 Challenges and future

In the field of CRC treatment, the combination of inducing chemotherapy and immunotherapy has emerged as a promising therapeutic strategy. However, there are still challenges and directions that need to be addressed in the future.

The TIME in CRC typically exhibits immunosuppressive features that limit the activity of immune cells. Future research can further explore the underlying mechanisms by which chemotherapy-induced immunotherapy overcomes this immune suppression, such as enhancing the activity of immune cells, modulating the polarization state of tumor-associated macrophages, disrupting tumor vasculature, and so on (87). To improve the success rate of chemotherapy-induced immunotherapy, it is crucial to explore innovative treatment targets/strategies and identify patients who respond better to specific treatment regimens.

scRNA-seq technology provides us with an opportunity to gain in-depth understanding of tumor and immune cell heterogeneity (209, 210). This technology can be used to reveal the roles of different cell subpopulations in chemotherapy-induced immunotherapy, thereby helping optimize treatment strategies and select the most suitable patients (211, 212). Combination therapies of chemotherapy and immunotherapy may yield better treatment outcomes compared to monotherapies. However, determining the optimal combination strategies and dosages remains challenging. Future research should focus on identifying the optimal drug combinations, timing of administration, dosages, and the best concentration-time curves in representative preclinical models (112, 213). Single-cell data can be utilized in future studies to achieve more precise treatment optimization. Other therapies such as photodynamic therapy, photothermal therapy, radiation therapy, and magnetic fluid hyperthermia can further induce ICD in tumor cells, enhance the efficacy of anti-tumor immunotherapy, expand their potential applications, and maximize clinical benefits (214).

Due to the limited predictive ability of current biomarkers such as PD-L1 expression and tumor mutation burden in cancer precision medicine, alternative biomarkers are still being explored (215, 216). A recent study has shown that advanced NSCLC patients with high PD-L1 expression and high immune infiltration can actually respond to PD-1 therapy plus chemotherapy in the first-line setting. For patients lacking PD-L1 expression or immune infiltration, chemotherapy may be a better treatment choice (217). This suggests that in the future, it is also significant to further explore alternative biomarkers in CRC, for guiding precision medicine in the clinical practice of CRC treatment.

Furthermore, due to significant biological differences among CRC patients, personalized immunotherapy approaches become crucial. scRNA-seq may reveal potential mechanisms regulating immune cell exhaustion and identify advanced biomarkers, thereby facilitating the design of novel personalized immunotherapy strategies (11, 218–220). By utilizing scRNA-seq to better understand individual variations, we can design optimal individualized chemotherapy-induced immunotherapy regimens for specific patients. These efforts are expected to provide more effective treatment choices and personalized treatment plans for CRC patients.

## 8 Conclusion

Immunotherapy has made significant progress in CRC, revolutionizing treatment outcomes. The TIME is closely related to tumor immunotherapy, which is a key obstacle to anti-tumor immunity and may limit the clinical benefits of immunotherapy. Recent studies have shown that some chemotherapy drugs can promote immune activation and enhance the efficacy of immunotherapy. By inducing ICD and exposing new antigens, it can activate CD8+ T cells and enhance the immune response to cancer. A large body of research has shown that most chemotherapy drugs exert immunostimulatory effects by inhibiting immune-suppressive cells or activating effector cells, or by increasing immunogenicity and T cells infiltration. inducing chemotherapy combined with immunotherapy has become a standard part of treatment in some tumors, and clinical trials have demonstrated its feasibility and safety in CRC. These combination therapies typically transform “cold” tumors that are insensitive to immune response into “hot” tumors. We propose a personalized exploration of inducing chemotherapy, gradually reducing chemotherapy regimens after systemic chemotherapy induction, combining with immunotherapy until only oral chemotherapy is maintained, and eventually transitioning to immune monotherapy maintenance treatment. In summary, chemotherapy-induced immunotherapy has enormous potential in the field of CRC. Future research will focus on overcoming the immune-suppressive microenvironment, applying scRNA-seq technology, achieving personalized treatment, researching predictive biomarkers, and optimizing combination therapy, among other challenges, to benefit more cancer patients in the near future.

## Author contributions

KJ and HL contributed to the conception and design of the study. SY drafted the initial manuscript and prepared the figures. YH and MY wrote sections of the manuscript. All authors participated in revising the manuscript, read, and approved the final version for submission.

## Glossary

**Table:**

ICD	immunogenic cell death
NM-neoAgs	non-mutated neoantigens
CRC	colorectal cancer
mCRC	metastatic CRC
mOS	median overall survival
ICIs	immune checkpoint inhibitors
TME	tumor microenvironment
TIME	tumor immune microenvironment
MSI-H	microsatellite instability
dMMR	mismatch repair-deficient
MSS	microsatellite stable
pMMR	proficient mismatch repair
DOX	doxorubicin
PTX	paclitaxel
OXA	oxaliplatin
DAMPs	danger-associated molecular patterns
NM-neoAgs	non-mutated neoantigens
CTL	cytotoxic T lymphocyte
scRNA-seq	single-cell RNA sequencing
PD-1/PD-L1	programmed death receptor 1/programmed death-ligand 1
FDA	Food and Drug Administration
Tregs	regulatory T cells
MDSCs	myeloid-derived suppressor cells
TAMs	tumor-associated macrophages
NK cells	natural killer cells
MHC I	major histocompatibility complex class I
TGF-β	transforming growth factor β
DCs	dendritic cells
5-fluorouracil	5-FU
EGFR	epidermal growth factor receptor
DAMPs	danger-associated molecular patterns
CRT	calreticulin
HMGB1	high mobility group box 1
mDCs	mature DCs
APCs	antigen-presenting cells
NSCLC	non-small cell lung cancer
GP	gemcitabine plus cisplatin
TAM1	tumor-associated macrophages type 1
TAM2	tumor-associated macrophages type 2
cGAS-STING	cyclic GMP-AMP synthase/stimulator of interferon genes
ICOS	inducible T cell co-stimulator
ILBs	innate-like B cells
T_H_ cells	helper T cells
IFN	interferon
TNBC	triple-negative breast cancer
HSP70	heat shock protein 70
PFS	progression-free survival
CTX	cyclophosphamide
MTX	methotrexate
SCLC	small cell lung cancer
SOC	standard of care
FTD/TPI	trifluridine/tipiracil
OS	overall survival
ASCO	American Society of Clinical Oncology
EFS	event-free survival
mPFS	median progression-free survival
ORR	overall response rate
ILB	innate immune B cell
T_F_H	follicular helper T cells
T_H_1	type 1 helper T cells
TAA	tumor-associated antigen
LARC	Locally advanced rectal cancer
